# Long Noncoding RNA lncCAMTA1 Promotes Proliferation and Cancer Stem Cell-Like Properties of Liver Cancer by Inhibiting CAMTA1

**DOI:** 10.3390/ijms17101617

**Published:** 2016-09-23

**Authors:** Li-Juan Ding, Yan Li, Shu-Dong Wang, Xin-Sen Wang, Fang Fang, Wei-Yao Wang, Peng Lv, Dong-Hai Zhao, Feng Wei, Ling Qi

**Affiliations:** 1 Department of Radio-Oncology, First Hospital of Jilin University, No. 71 Xinmin Street, Changchun 130021, China; dinglijuanjl@163.com; 2 Academy of Laboratory, Jilin Medical University, No. 5 Jilin Street, Jilin 132013, China; yanli1986jl@sina.com (Y.L.); fangjifang2@sina.com (F.F.); 3 Center of Cardiovascular Diseases, First Hospital of Jilin University, No. 71 Xinmin Street, Changchun 130021, China; wangshudongjl@163.com; 4 Department of Interventional Therapy, First Hospital of Jilin University, No. 71 Xinmin Street, Changchun 130021, China; xinsenwang3@126.com; 5 Department of Pathology, Jilin Medical University, No. 5 Jilin Street, Jilin 132013, China; wangweiyao2@sina.cn (W.-Y.W.); penglv32@sina.cn (P.L.); zhaodong_hai@sina.com (D.-H.Z.); 6 Department of Hepatobiliary and Pancreas Surgery, First Hospital of Jilin University, No. 71 Xinmin Street, Changchun 130021, China

**Keywords:** long noncoding RNA, cancer stem cells, liver cancer, tumorigenesis, CAMTA1

## Abstract

Hepatocellular carcinoma (HCC) is the most common subtype of liver malignancy, and it is characterized by poor prognosis because of cancer stem cell (CSC)-mediated high postsurgical recurrence rates. Thus, targeting CSCs, or HCC cells with CSC-like properties, is an effective strategy for HCC therapy. Here, using long noncoding RNA (lncRNA) microarray analysis, we identified a novel lncRNA termed lncCAMTA1 that is increased in both liver CSCs and HCC. High lncCAMTA1 expression in HCC indicates poor clinical outcome. In vitro and in vivo functional experiments showed that overexpression of lncCAMTA1 promotes HCC cell proliferation, CSC-like properties, and tumorigenesis. Conversely, depletion of lncCAMTA1 inhibits HCC cell proliferation, CSC-like properties, and tumorigenesis. Mechanistically, we demonstrated that lncCAMTA1 physically associates with the *calmodulin binding transcription activator 1* (*CAMTA1*) promoter, induces a repressive chromatin structure, and inhibits CAMTA1 transcription. Furthermore, CAMTA1 is required for the effects of lncCAMTA1 on HCC cell proliferation and CSC-like properties, and the expression of lncCAMTA1 and CAMTA1 is significantly negatively correlated in HCC tissues. Collectively, our study revealed the important roles and underlying molecular mechanisms of lncCAMTA1 on HCC, and suggested that lncCAMTA1 could be an effective prognostic factor and a potential therapeutic target for HCC.

## 1. Introduction

Liver cancer is the fifth most common cancer and the second highest cause of cancer-related death in men worldwide [1]. Annually, about 780,000 new cases of liver cancer are diagnosed [2]. Hepatocellular carcinoma (HCC) is the major histological subtype and accounts for 78% of liver cancer [3]. Current standard treatments such as surgical resection, liver transplantation, and sorafenib only provide limited survival benefits due to high postsurgical recurrence rates [4,5]. About 16.6% of people survive for 5 years after being diagnosed with liver cancer [2]. It is of paramount importance to clarify the underlying molecular mechanisms of HCC tumorigenesis and progression, and to develop novel diagnostic and therapeutic strategies for HCC [6,7,8].

The high postsurgical recurrence rates are mainly ascribed to the heterogeneous population of HCC cells including a subset of cancer stem cells (CSCs), which have the capacity to self-renew, differentiate, and give rise to a new cancer [9,10,11]. They are considered to account for the initiation, progression, metastasis and relapse of cancers [12]. Liver cancer stem cells (LCSCs) have been identified in many previous studies and could be enriched with various markers, including EpCAM, CD133, CD13, CD24, CD90, OV6, etc. [13,14,15,16] Although many reports have shown that various stemness-related transcription factors and signaling pathways are involved in the self-renewal of LCSCs and the CSC-like properties of HCC cells [17,18], the involvement of long noncoding RNAs remains largely unknown.

Long noncoding RNAs (lncRNAs) are a novel class of RNA transcripts longer than 200 nucleotides in length, yet with no protein-coding potential [19,20,21]. Thus far, accumulating evidences demonstrate that lncRNAs regulate a wide range of important pathophysiological processes, particularly in cancers, such as cell growth, cell cycle, cell apoptosis, cell migration, drug resistance, and others [22,23,24]. Notably, many lncRNAs have been found to be aberrantly expressed in multiple cancers, including HCC [25,26,27]. These aberrantly expressed lncRNAs could regulate gene expression in *cis* or in *trans* by diverse mechanisms [28,29,30]. Particularly, one of the important regulation mechanisms of lncRNAs is to physically associate with chromatin or chromatin modifiers such as HOX Transcript Antisense RNA (HOTAIR), SWI/SNF Complex Antagonist Associated With Prostate Cancer 1 (SChLAP1), lncTCF7, and Khps1 to change chromatin structure and gene transcription [9,31,32,33]. However, the potential roles and regulation mechanisms of lncRNAs in the CSC-like properties of HCC cells are largely unclear.

In this study, combining LCSC microarray results [34] with two human HCC microarray results (GSE58043 and GSE55191), we identified an intriguing lncRNA, termed lncCAMTA1 (gene symbol RP11-312B8.1), whose expression is upregulated in both LCSCs and HCC. We further verified the aberrant expression of lncCAMTA1 in LCSCs and HCC, and evaluated its association with the prognosis of HCC patients. Moreover, using in vitro and in vivo functional experiments, we investigated the roles and underlying molecular mechanisms of lncCAMTA1 on proliferation, CSC-like properties, and tumorigenesis of HCC cells.

## 2. Results

### 2.1. lncCAMTA1 Expression Pattern in Public Database of LCSC and HCC

**Figure 1:**
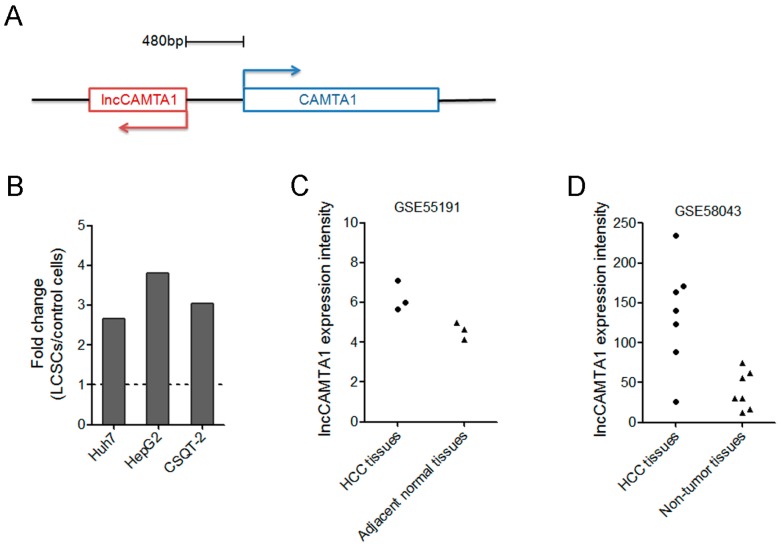
lncCAMTA1 expression pattern in public database of liver cancer stem cells (LCSCs) and hepatocellular carcinoma (HCC). (**A**) Schematic illustration of the genomic organization of lncCAMTA1 and calmodulin binding transcription activator 1 (CAMTA1). Arrows indicate transcription direction; (**B**) lncCAMTA1 is upregulated in LCSCs in comparison with their respective control cells; (**C**) lncCAMTA1 is upregulated in HCC tissues from GSE55191 data; (**D**) lncCAMTA1 is upregulated in HCC tissues from GSE58043 data.

Many lncRNAs differentially expressed in LCSCs were identified by microarray in a previous report [34]. To further identify LCSC-associated lncRNAs deregulated in HCC, we compared the LCSC microarray results with two human HCC microarray results (GSE58043 and GSE55191). Among these differentially expressed lncRNAs both in LCSCs and HCC, we focused on an uncharacterized lncRNA, termed lncCAMTA1 (gene symbol RP11-312B8.1). lncCAMTA1 resides on chromosome 1 and orients in antisense direction with respect to calmodulin binding transcription activator 1 (CAMTA1), a critical tumor suppressor in many cancers [35,36,37] (Figure 1A). Microarray results showed that lncCAMTA1 is consistently upregulated in all LCSCs (Figure 1B). Moreover, lncCAMTA1 is increased in HCC tissues in comparison with non-tumor tissues in both HCC lncRNA microarray datasets (Figure 1C,D). The full-length sequence of lncCAMTA1 was confirmed by 5′ and 3′ rapid amplification of cDNA ends (RACE) analyses (Figure S1A). Cellular fractionation assays showed that lncCAMTA1 was mainly localized in the nucleus of HCC cells (Figure S1B).

### 2.2. lncCAMTA1 Is Highly Expressed in LCSCs

**Figure 2:**
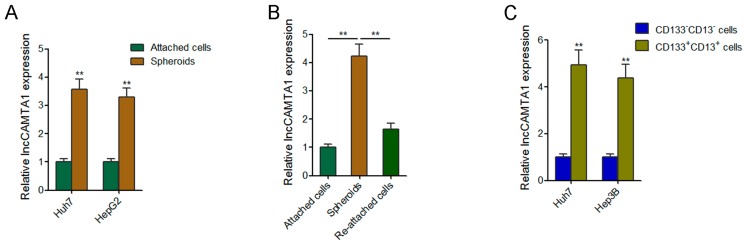
lncCAMTA1 is highly expressed in LCSCs. (**A**) LncCAMTA1 is upregulated in the spheroids compared with the attached cells derived from Huh7 and HepG2 cells; (**B**) lncCAMTA1 is upregulated in the spheroids derived from HCCLM3 cells, and partially restored during reattachment; (**C**) lncCAMTA1 is upregulated in CD133^+^CD13^+^ cells compared with CD133^−^CD13^−^ cells derived from Huh7 and Hep3B cells. Data are shown as mean ± standard deviation (SD) from at least three independent experiments. ** *p* < 0.01 by Student’s *t* test.

To confirm the expression pattern of lncCAMTA1 in LCSCs, we enriched LCSCs through inducing hepatoma spheroid formation and examined lncCAMTA1 expression in the self-renewing spheroids and the attached cells. As shown in Figure 2A, lncCAMTA1 is significantly more highly expressed in the spheroids than that in the attached cells derived from both Huh7 and HepG2 cells. Moreover, the increased expression of lncCAMTA1 was partially restored during reattachment in HCCLM3 cells (Figure 2B). Furthermore, increased expression of lncCAMTA1 was detected in CD133^+^CD13^+^ cells compared with CD133^−^CD13^−^ cells derived from Huh7 and Hep3B cells (Figure 2C). Collectively, these results confirmed the increased expression of lncCAMTA1 in LCSCs.

### 2.3. lncCAMTA1 Is Increased in HCC and Indicates Poor Outcome of HCC Patients

**Figure 3:**
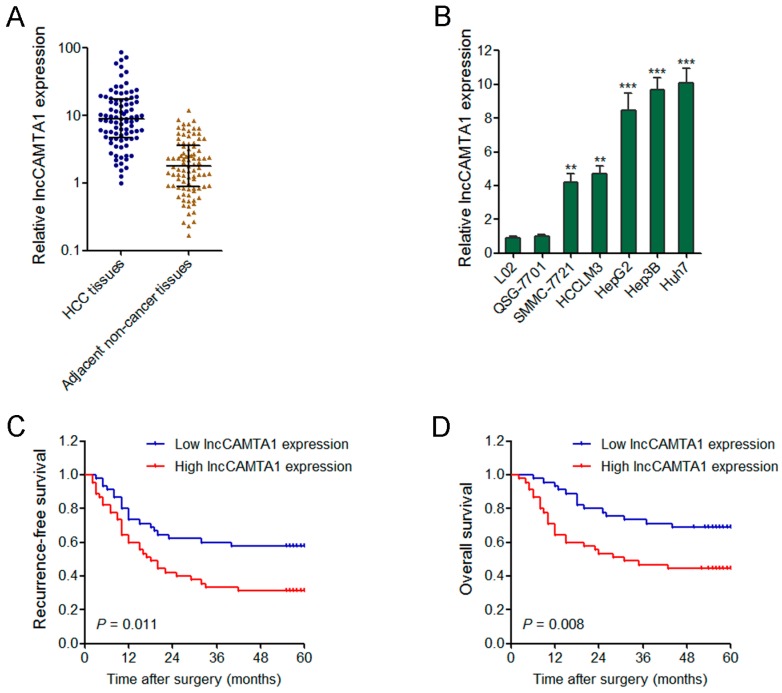
lncCAMTA1 is increased in HCC and indicates poor outcome. (**A**) lncCAMTA1 is increased in HCC tissues in comparison with paired adjacent non-cancerous hepatic tissues. Data are shown as median with interquartile range. *n* = 90, *p* < 0.001 by Wilcoxon signed-rank test; (**B**) lncCAMTA1 is increased in HCC cell lines SMMC-7721, MHCC97H, HCCLM3 and HepG2, in comparison with normal liver cell lines L02 and QSG-7701. Data are shown as mean ± SD from at least three independent experiments. ** *p* < 0.01, *** *p* < 0.001 by Student’s *t* test; (**C**,**D**) Kaplan–Meier analyses indicated that patients with high expression of lncCAMTA1 had a shorter recurrence-free survival (*p* = 0.011, log-rank test); (**C**) and overall survival (*p* = 0.008, log-rank test); (**D**) time than those with low expression of lncCAMTA1.

To further confirm the expression pattern of lncCAMTA1 in HCC, we measured lncCAMTA1 expression in 90 pairs of HCC tissues and adjacent non-cancerous hepatic tissues by quantitative real-time PCR (qRT-PCR). Our results showed that lncCAMTA1 is significantly increased in HCC tissues compared with adjacent non-cancerous tissues (Figure 3A). We further measured the expression of lncCAMTA1 in normal liver cell lines L02 and QSG-7701, and HCC cell lines SMMC-7721, HCCLM3, HepG2, Hep3B, and Huh7. The results showed that lncCAMTA1 is also significantly upregulated in HCC cells compared with normal liver cells (Figure 3B).

We next evaluated the prognostic value of lncCAMTA1 for HCC patients. Kaplan–Meier survival analyses showed that patients with higher lncCAMTA1 levels possessed a worse recurrence-free survival and overall survival after surgical resection (Figure 3C,D). Collectively, these results suggested that high lncCAMTA1 expression indicates poor outcome of HCC patients, and lncCAMTA1 might act as an oncogene in HCC.

### 2.4. Overexpression of lncCAMTA1 Promotes HCC Cell Proliferation and CSC-Like Properties in Vitro, and Tumorigenesis in Vivo

**Figure 4:**
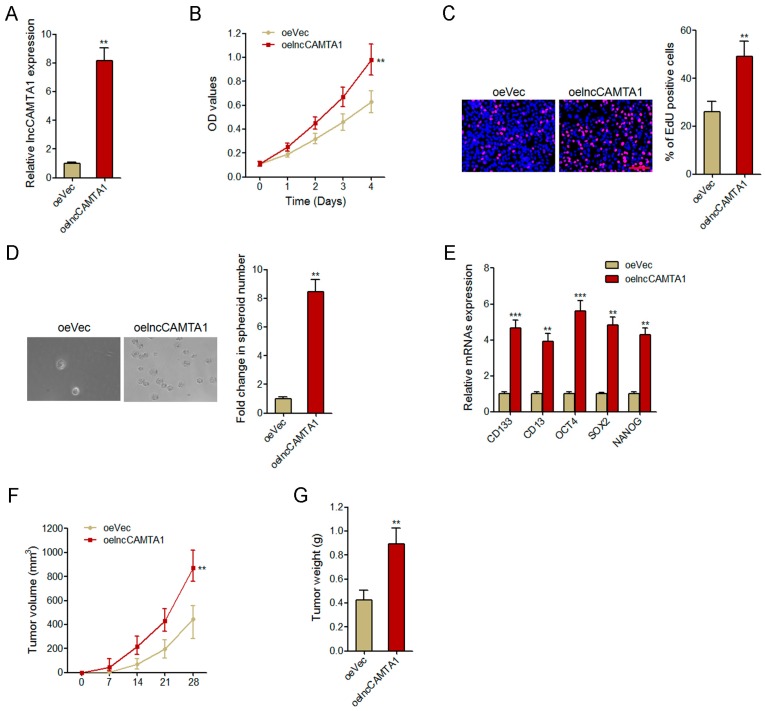
Overexpression of lncCAMTA1 promotes HCC cell proliferation, CSC-like properties, and tumorigenesis. (**A**) lncCAMTA1 expression in HCCLM3 cells stably overexpressing lncCAMTA1 and control HCCLM3 cells; (**B**) Cell proliferation of HCCLM3 cells stably overexpressing lncCAMTA1 and control HCCLM3 cells was detected by cell counting kit-8 (CCK-8) assay, and the relative number of cells compared to day 0 is presented; (**C**) Cell proliferation of HCCLM3 cells stably overexpressing lncCAMTA1 and control HCCLM3 cells was detected by 5-ethynyl-2′-deoxyuridine (EdU) incorporation assay. Scale bars = 100 μm; (**D**) Spheroid formation assays showed that overexpression of lncCAMTA1 increased the number of spheroids generated from HCCLM3 cells; (**E**) Overexpression of lncCAMTA1 upregulated the expression of stem cell markers and transcription factors CD133, CD13, OCT4, SOX2, and NANOG. For **A**–**E**, data are shown as mean ± SD. ** *p* < 0.01 by Student’s *t* test, *** *p* < 0.001 by Student’s *t* test; (**F**,**G**) Overexpression of lncCAMTA1 promoted in vivo tumor growth of HCCLM3 cells. HCCLM3 cells stably overexpressing lncCAMTA1 and control HCCLM3 cells were subcutaneously injected into nude mice. Tumor volumes were measured every 7 days (**F**), and tumor weights were measured at 28 days after injection (**G**). *n* = 6 mice in each group, ** *p* < 0.01 by Mann–Whitney test. oeVec: empty vector control overexpression; oelncCAMTA1; lncCAMTA1 overexpression; OD: optical density.

To explore the functions of lncCAMTA1 in HCC, we stably overexpressed lncCAMTA1 in HCCLM3 cells via transfecting lncCAMTA1 overexpression plasmids (Figure 4A). Cell counting kit-8 (CCK-8) assays and 5-ethynyl-2′-deoxyuridine (EdU) incorporation assays were performed for the detection of cell proliferation. Our results showed that overexpression of lncCAMTA1 significantly promoted HCCLM3 cell proliferation (Figure 4B,C). Spheroid formation assays were performed to examine the CSC-like properties of HCC cells. As shown in Figure 4D, spheroid formation was significantly enhanced in HCCLM3 cells overexpressing lncCAMTA1. Overexpression of lncCAMTA1 also resulted in a significant increase of stem cell markers and transcription factors CD133, CD13, OCT4, SOX2, and NANOG (Figure 4E). To evaluate the effects of lncCAMTA1 on HCC tumorigenesis in vivo, we injected HCCLM3 cells stably overexpressing lncCAMTA1 and control HCCLM3 cells subcutaneously into nude mice for xenograft. The results showed that overexpression of lncCAMTA1 significantly promoted xenografted tumor growth, tumor size and tumor weight in vivo (Figure 4F,G). These data suggested that overexpression of lncCAMTA1 promotes HCC cell proliferation, CSC-like properties and tumorigenesis.

### 2.5. Depletion of lncCAMTA1 Inhibits HCC Cell Proliferation and CSC-Like Properties in Vitro, and Tumorigenesis in Vivo

**Figure 5:**
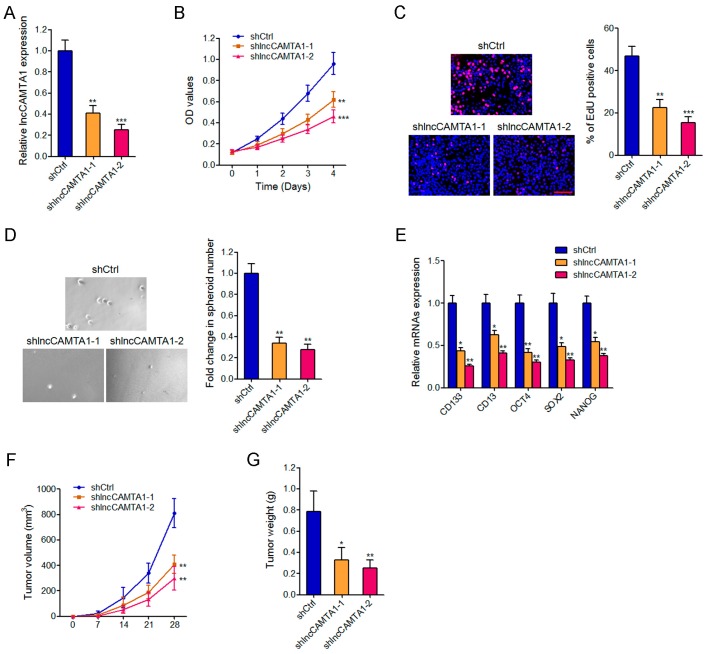
Depletion of lncCAMTA1 inhibits HCC cell proliferation, CSC-like properties, and tumorigenesis. (**A**) lncCAMTA1 expression in lncCAMTA1 stably depleted and control HepG2 cells; (**B**) Cell proliferation of lncCAMTA1 stably depleted and control HepG2 cells was detected by CCK-8 assay, and the relative number of cells compared to day 0 is presented; (**C**) Cell proliferation of lncCAMTA1 stably depleted and control HepG2 cells was detected by EdU incorporation assays. Scale bar = 100 μm; (**D**) Spheroid formation assays showed that knockdown of lncCAMTA1 decreased the number of spheroids generated from HepG2 cells; (**E**) Depletion of lncCAMTA1 downregulated the expression of stem cell markers and transcription factors CD133, CD13, OCT4, SOX2, and NANOG. For **A**–**E**, data are shown as mean ± SD. * *p* < 0.05, ** *p* < 0.01, *** *p* < 0.001 by Student’s *t* test; (**F**,**G**) Depletion of lncCAMTA1 inhibited in vivo tumor growth of HepG2 cells. lncCAMTA1 stably depleted and control HepG2 cells were subcutaneously injected into nude mice. Tumor volumes were measured every 7 days (**F**), and tumor weights were measured at 28 days after injection (**G**). *n* = 6 mice in each group, * *p* < 0.05, ** *p* < 0.01 by Mann–Whitney test.

To further characterize the roles of lncCAMTA1 in HCC, we stably knocked down lncCAMTA1 in HepG2 cells by transfecting small hairpin RNAs (shRNAs) specifically targeting lncCAMTA1. Two independent shlncCAMTA1 exhibited effective reduction of lncCAMTA1 transcript levels and were used in subsequent experiments (Figure 5A). CCK-8 assays and EdU incorporation assays showed that depletion of lncCAMTA1 significantly repressed HepG2 cell proliferation (Figure 5B,C). Spheroid formation was significantly decreased in lncCAMTA1-depleted HepG2 cells (Figure 5D). Consistently, depletion of lncCAMTA1 also resulted in a significant decrease of stem cell markers and transcription factors CD133, CD13, OCT4, SOX2, and NANOG (Figure 5E). lncCAMTA1 stably depleted and control HepG2 cells were subcutaneously injected into nude mice for xenograft. The results showed that depletion of lncCAMTA1 significantly inhibited xenografted tumor growth, tumor size and tumor weight in vivo (Figure 5F,G). Collectively, these results revealed that depletion of lncCAMTA1 represses HCC cell proliferation, CSC-like properties and tumorigenesis.

### 2.6. lncCAMTA1 Inhibits CAMTA1 Expression via Changing Chromatin Structure at the CAMTA1 Promoter

**Figure 6:**
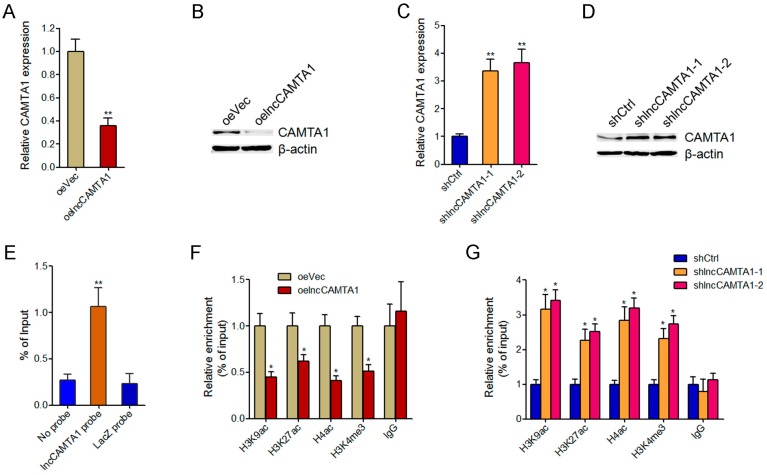
lncCAMTA1 inhibits CAMTA1 expression via changing chromatin structure at the CAMTA1 promoter. (**A**) CAMTA1 mRNA levels in HCCLM3 cells stably overexpressing lncCAMTA1 and control HCCLM3 cells; (**B**) CAMTA1 protein levels in HCCLM3 cells stably overexpressing lncCAMTA1 and control HCCLM3 cells; (**C**) CAMTA1 mRNA levels in lncCAMTA1 stably depleted and control HepG2 cells; (**D**) CAMTA1 protein levels in lncCAMTA1 stably depleted and control HepG2 cells; (**E**) Chromatin isolation by RNA purification (ChIRP) assays showed that lncCAMTA1 has a significantly genomic occupancy on the CAMTA1 promoter; (**F**,**G**) Chromatin immunoprecipitation (ChIP) assays were performed using H3K9ac, H3K27ac, H4ac, H3K4me3, or non-specific IgG antibodies in HCCLM3 cells stably overexpressing lncCAMTA1 or control HCCLM3 cells (**F**), lncCAMTA1 stably depleted or control HepG2 cells (**G**). The retrieved DNA was amplified by quantitative PCR (qPCR) to measure the occupancy of indicated histone marks at the CAMTA1 promoter. For all panels, data are shown as mean ± SD. * *p* < 0.05, ** *p* < 0.01 by Student’s *t* test.

As CAMTA1 has been reported to be a critical tumor suppressor in many cancers, and also because of its nearby genomic location to lncCAMTA1, we first evaluated whether lncCAMTA1 could regulate CAMTA1 expression in HCC cells. Our results showed that overexpression of lncCAMTA1 in HCCLM3 cells significantly decreased CAMTA1 mRNA and protein levels (Figure 6A,B). In contrast, knockdown of lncCAMTA1 in HepG2 cells significantly upregulated CAMTA1 mRNA and protein levels (Figure 6C,D). Previous studies have reported that many nuclear lncRNAs could directly bind to genomic DNA and change chromatin structure and gene expression [33,38]. To test whether lncCAMTA1 regulates CAMTA1 expression through a similar mechanism, we performed chromatin isolation by RNA purification (ChIRP) assays to determine whether lncCAMTA1 could directly bind to the CAMTA1 promoter. ChIRP assays showed that lncCAMTA1 has a significant genomic occupancy on the CAMTA1 promoter (Figure 6E). Furthermore, chromatin immunoprecipitation (ChIP) assays showed that overexpression of lncCAMTA1 significantly decreased euchromatic histone marks at the CAMTA1 promoter (Figure 6F), and depletion of lncCAMTA1 significantly increased euchromatic histone marks at the CAMTA1 promoter (Figure 6G). Collectively, these results suggested that lncCAMTA1 inhibits CAMTA1 expression via changing chromatin structure at the CAMTA1 promoter.

### 2.7. lncCAMTA1 Transcript Level Was Negatively Correlated with CAMTA1 mRNA Level in HCC Tissues

**Figure 7:**
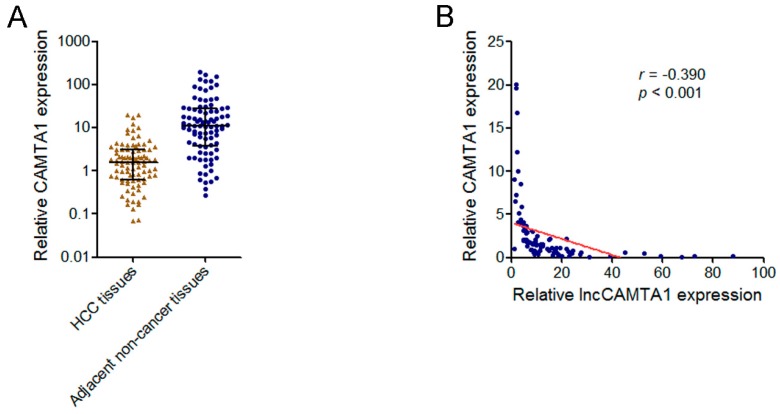
lncCAMTA1 expression is negatively correlated with CAMTA1 in HCC tissues. (**A**) CAMTA1 expression in HCC tissues and adjacent non-cancerous hepatic tissues. Data are shown as median with interquartile range. *n* = 90, *p* < 0.001 by Wilcoxon signed-rank test; (**B**) lncCAMTA1 and CAMTA1 expression levels were negatively correlated in HCC tissues. *r* = −0.390, *p* < 0.001 by Pearson correlation analysis.

Because lncCAMTA1 inhibits CAMTA1 expression, we next evaluated whether a correlation exists between CAMTA1 and lncCAMTA1 expression level in HCC tissues. We measured CAMTA1 mRNA level in the same HCC tissues and adjacent non-cancerous tissues used in Figure 3A. As shown in Figure 7A, CAMTA1 mRNA level was significantly downregulated in HCC tissues compared with paired adjacent non-cancerous tissues. Next, we performed a correlation analysis of CAMTA1 and lncCAMTA1 expression level in the 90 HCC tissues. As shown in Figure 7B, CAMTA1 expression was negatively correlated with lncCAMTA1 transcript level (*r* = −0.390, *p* < 0.001, Pearson’s correlation), supporting the inhibitory role of lncCAMTA1 on CAMTA1.

### 2.8. CAMTA1 Is Required for the Effects of lncCAMTA1 on HCC Cell Proliferation and CSC-Like Properties

**Figure 8:**
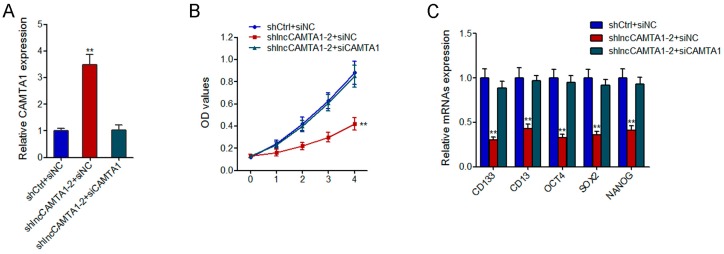
CAMTA1 is required for the effects of lncCAMTA1 on HCC cell proliferation and stemness properties. (**A**) CAMTA1 expression in lncCAMTA1 stably depleted and control HepG2 cells; (**B**) CCK-8 assays showed that knockdown of CAMTA1 abrogated the proliferation-inhibitory effect of lncCAMTA1 depletion on HepG2 cells; (**C**) Knockdown of CAMTA1 abrogated the decrease of stem cell markers and transcription factors CD133, CD13, OCT4, SOX2, and NANOG caused by lncCAMTA1 depletion. Data are shown as mean ± SD. ** *p* < 0.01 by Student’s *t* test.

To investigate whether the effects of lncCAMTA1 on the proliferation and CSC-like properties of HCC cells are dependent on CAMTA1, we knocked down CAMTA1 in lncCAMTA1 stably depleted and control HepG2 cells (Figure 8A). CCK-8 assays showed that knockdown of CAMTA1 abrogated the proliferation-inhibitory effect of lncCAMTA1 depletion on HepG2 cells (Figure 8B). Knockdown of CAMTA1 also abrogated the decrease of stem cell markers and transcription factors CD133, CD13, OCT4, SOX2, and NANOG caused by lncCAMTA1 depletion (Figure 8C). These results suggested that CAMTA1 is required for the effects of lncCAMTA1 on HCC cell proliferation and CSC-like properties.

## 3. Discussion

Liver cancer represents one of the most common cancers and the second highest cause of cancer-related death worldwide [1]. Despite the fact that surgical resection and liver transplantation have sustained patients’ survival, the overall outcome of HCC patients remains disappointing [2]. Recently, the identification of CSCs in many malignancies, including HCC, has renovated our view of malignant tumors [10]. CSCs, also known as tumor initiating cells, have stemness characteristics such as self-renewal and differentiation [39]. They have the capacity to form neoplastic and metastatic tumors, and may account for the high postsurgical recurrence rates [40]. Therefore, a continuous research for effectively eliminating HCC cells with CSC-like characteristics is critical.

In the current study, using published lncRNA microarray results of LCSCs and HCC [34], we found lncCAMTA1 is consistently upregulated in both LCSCs and HCC. We further confirmed the aberrant expression of lncCAMTA1 in LCSCs and a cohort containing 90 pairs of HCC tissues and adjacent non-cancerous hepatic tissues. In comparison with normal liver cells, lncCAMTA1 was also highly expressed in HCC cells. In clinical HCC samples, using Kaplan–Meier analysis, we found that lncCAMTA1 is associated with poor outcome of HCC patients. The previously reported results and our own results collectively confirmed the aberrant expression of lncCAMTA1 in LCSCs and HCC, and suggested that lncCAMTA1 may be a novel prognostic biomarker for HCC.

In vitro and in vivo functional experiments demonstrated that overexpression of lncCAMTA1 significantly promoted HCC cell proliferation and CSC-like properties in vitro, and tumorigenesis in vivo. In contrast, depletion of lncCAMTA1 significantly inhibited HCC cell proliferation and CSC-like properties in vitro, and tumorigenesis in vivo. Mechanistically, we found that lncCAMTA1 physically associated with the CAMTA1 promoter, induced a suppressive chromatin structure, and inhibited CAMTA1 transcription. CAMTA1 has been reported as a tumor suppressor, which inhibits proliferation and activates differentiation programs in many cancer cells and cancer stem cells [35,36]. In this study, we also found that depletion of CAMTA1 promoted HCC cell proliferation and CSC-like properties. More importantly, the depletion of CAMTA1 abrogated the effects of lncCAMTA1 on HCC cell proliferation and CSC-like properties. CAMTA1 expression was negatively correlated with lncCAMTA1 transcript level in HCC tissues, supporting the inhibitory role of lncCAMTA1 on CAMTA1. In summary, these data demonstrated that CAMTA1 is a critical downstream mediator of the effects of lncCAMTA1 on HCC cell proliferation and CSC-like properties.

In addition to lncCAMTA1, several lncRNAs have been reported to regulate the self-renewal and tumorigenesis of LCSCs. lncTCF7 enhances the self-renewal and tumorigenic capacity of LCSCs via activating Wnt signaling [9]. lncRNA DILC inhibits LCSC expansion and HCC initiation and progression [34]. lncRNA PVT1 promotes stem cell-like properties of HCC cells through stabilizing NOP2 [27]. Our new data in lncCAMTA1, combined with these reports, revealed the important roles and the complex and diverse molecular mechanisms of lncRNAs in LCSCs and HCC.

In conclusion, our data demonstrated that lncCAMTA1 promotes HCC cell proliferation and CSC-like properties through inhibiting CAMTA1, and suggested that lncCAMTA1 could be an effective prognostic biomarker and therapeutic target for HCC.

## 4. Materials and Methods

### 4.1. Microarray Data Analysis

Microarray data of LCSCs were acquired from Supplementary Table 7 of Wang et al. [34]. The GenBank accession number of lncCAMTA1 is ENST00000442889. Microarray data of human HCC were downloaded from Gene Expression Omnibus (GEO) under accession numbers GSE58043 and GSE55191. The ID_REF for lncCAMTA1 in GSE58043 was 51510, and in GSE55191 it was ASHG19A3A051901.

### 4.2. Patients and Sample Collection

In total, 90 pairs of fresh HCC tissues and paired adjacent non-cancerous hepatic tissues were obtained from hepatectomy of HCC patients without preoperative treatments at the First Hospital of Jilin University (Changchun, China). All the tissues were histologically and pathologically examined by two experienced pathologists. The Ethics Committee of the First Hospital of Jilin University (Ethical Approval No. 2014-018; Date 15 April 2014) reviewed and approved this study. All patients signed informed consent.

### 4.3. Cell Culture

The normal human liver cell lines L02 and QSG-7701, and human HCC cell lines SMMC-7721, HCCLM3, HepG2, Hep3B, and Huh7 were obtained from the Shanghai Institute of Biochemistry and Cell Biology, Chinese Academy of Sciences (Shanghai, China). All the cells were cultured in Dulbecco’s modified Eagle’s medium (DMEM) supplemented with 10% fetal bovine serum (FBS) (Gibco, New York, NY, USA) in a humidified 5% CO_2_ incubator (Thermo-Fisher Scientific, Waltham, MA, USA) at 37 °C.

### 4.4. Flow Cytometry

Cells were stained with phycoeritrin (PE)-conjugated anti-human CD133 and fluorescein isothiocyanate (FITC)-conjugated anti-human CD13 antibodies (eBioscience, San Diego, CA, USA), followed by sorting using a FACSCalibur (BD Immunocytometry Systems, San Jose, CA, USA) as previously described [41].

### 4.5. 5′ and 3′ Rapid Amplification of cDNA Ends

5′ RACE and 3′ RACE experiments were performed to confirm the transcriptional initiation and termination sites of lncCAMTA1 using a SMARTer™ RACE cDNA Amplification Kit (Clontech, Palo Alto, CA, USA) following the manufacturer’s protocols. The gene specific primers were as follows:

5′ RACE, 5′–TGCCAGAACACTTGAACGAGGATTTTTC–3′;
3′ RACE, 5′–GAAAAATCCTCGTTCAAGTGTTCTGGC–3′.

### 4.6. Cytoplasmic and Nuclear RNA Isolation

The Cytoplasmic and Nuclear RNA Purification Kit (Norgen, Belmont, CA, USA) was used to perform cytoplasmic and nuclear RNA isolation following the manufacturer’s protocols.

### 4.7. RNA Extraction and Quantitative Real-Time Polymerase Chain Reaction

Total RNA was extracted with the TRIzol reagent (Takara, Dalian, China) according to the manufacturer’s protocols. First-strand cDNA was generated using the M-MLV Reverse Transcriptase (Invitrogen, Carlsbad, CA, USA). qRT-PCR was performed using a SYBR Green PCR Kit (Takara) on ABI Prism 7500 (Applied Biosystems, Foster City, CA, USA). The gene specific primers were as follows: 5′-GGAAAGAAAAGTTGAATC-3′ (forward) and 5′-TCTGGAAAACCTAAGACA-3′ (reverse) for lncCAMTA1; 5′-AATCGTGCGTGACATTAAGGAG-3′ (forward) and 5′-ACTGTGTTGGCGTACAGGTCTT-3′ (reverse) for β-actin; 5′-TGTCACTGTCTTGTACCCTTG-3′ (forward) and 5′-TTCCTGTGGGCGGATTAG-3′ (reverse) for U6; 5′-AGTCGGAAACTGGCAGATAGC-3′ (forward) and 5′-GGTAGTGTTGTACTGGGCCAAT-3′ (reverse) for CD133; 5′-GACCAAAGTAAAGCGTGGAATCG-3′ (forward) and 5′-TCTCAGCGTCACCCGGTAG-3′ (reverse) for CD13; 5′-CATGGCTTAGAAGTGGAAAT-3′ (forward) and 5′-TTGGTGTTGCTGGTGAGT-3′ (reverse) for OCT4; 5′-CTGGGTTGATCCTCGGACCT-3′ (forward) and 5′-CCATCGGAGTTGCTCTCCA-3′ (reverse) for SOX2; 5′-AATACCTCAGCCTCCAGCAGATG-3′ (forward) and 5′-TGCGTCACACCATTGCTATTCTTC-3′ (reverse) for NANOG; 5′-ATGGCTAACAGAGAGGTGG-3′ (forward) and 5′-CAGGTGTGGCTTCAATAATG-3′ (reverse) for CAMTA1; 5′-GGAGCGAGATCCCTCCAAAAT-3′ (forward) and 5′-GGCTGTTGTCATACTTCTCATGG-3′ (reverse) for GAPDH. GAPDH was used as an endogenous control.

### 4.8. Vectors and Stable Cell Lines Construction

lncCAMTA1 cDNA was PCR amplified using the TaKaRa Ex Taq^®^ Hot Start Version DNA Polymerase (Takara) and subcloned into the BamH I and Xba I sites of pcDNA3.1(+) vector. The primers’ sequences were as follows: 5′-CGGGATCCGCGGGGGGCCTCGGG-3′ (sense) and 5′-GCTCTAGACTTTTGGGACATCTTTATTTCTGG-3′ (antisense). The constructed vector and pcDNA3.1 were transfected into HCCLM3 cells, and selected with neomycin (1000 μg/mL) for four weeks.

Two individual shRNAs targeting lncCAMTA1 were designed and synthesized. The shlncCAMTA1-1 sequence was 5′-ATCCTCGTTCAAGTGTTCT-3′ and the shlncCAMTA1-2 sequence was 5′-GACTGTCACACTGAATTCT-3′. After annealing, the oligonucleotides were inserted in the SuperSilencing™ shRNA expression vector pGPH1/Neo (GenePharma, Shanghai, China). Scrambled shRNA was used as negative control. The lncCAMTA1 specific or scrambled shRNA expression vectors were transfected into HepG2 cells and selected with neomycin (1000 μg/mL) for four weeks.

### 4.9. Small Interfering RNA (siRNA) Synthesis and Transfection

A pool of siRNAs specifically targeting CAMTA1 was purchased from Dharmacon (ON-TARGETplus Human CAMTA1 siRNA, Lafayette, CO, USA). Transfection was performed using Lipofectamine 3000 (Invitrogen) following the manufacturer’s protocols.

### 4.10. Western Blotting

The total cellular proteins were retrieved from indicated HCC cells using radio immunoprecipitation assay (RIPA) buffer (Beyotime, Shanghai, China) containing protease inhibitors. Equal amounts of proteins were separated by 10% sodium dodecyl sulfate-polyacrylamide gel electrophoresis and transferred to 0.22 μm nitrocellulose membrane (Millipore, Bedford, MA, USA). After blocking with 5% bovine serum albumin (BSA), the membrane was incubated with CAMTA1 (sc-368212, 1:500, Santa Cruz Biotechnology, Santa Cruz, CA, USA) or β-actin (ab1801, 1:1000, Abcam, Hong Kong, China) specific primary antibodies. Then the membranes were washed with Tris-buffered saline with Tween (TBST), followed by incubating with IRdye 800-conjugated goat anti-rabbit IgG (Abcam, Hong Kong, China), and detected using an Odyssey infrared scanner (Li-Cor Biosciences, Lincoln, NE, USA).

### 4.11. Cell Proliferation Assay

Cell proliferation was detected using CCK-8 (Dojindo Laboratories, Kumamoto, Japan) assays and EdU incorporation assays. For CCK-8 assays, equal numbers of indicated HCC cells were seeded in 96-well plates, and the absorbance at 450 nm was measured every day following the manufacturer’s instructions. For EdU incorporation assays, an EdU Kit (Roche, Mannheim, Germany) was used following the manufacturer’s protocols.

### 4.12. Spheroid Formation Assay

Indicated HCC cells were plated on ultra-low attachment culture dishes (Corning, Tewksbury, MA, USA) and cultured in DMEM/F12 (Gibco, New York, NY, USA) supplemented with 1% FBS, 20 ng/mL epithelial growth factor, and 20 ng/mL fibroblast growth factor for two weeks. The number of spheroids was counted under a stereomicroscope (Olympus, Tokyo, Japan).

### 4.13. Xenografted Tumor Growth

The Animal Care and Use Committee of the First Hospital of Jilin University (Ethical Approval No.2014-018; Date 15 April 2014) approved the animal experiments. Male athymic BALB/c nude mice used in this study were purchased from the Experimental Animal Center of the Chinese Academy of Sciences (Shanghai, China). 2 × 10^6^ indicated HCC cells were subcutaneously injected into the mice. Tumor growth was measured every 7 days with a caliper, and tumor volume was calculated as a × b^2^/2 (a, long axes; b, short axes).

### 4.14. Chromatin Isolation by RNA Purification Assay

The ChIRP assay was performed with the Magna ChIRP RNA Interactome Kit (Millipore) according to the manufacturer’s protocols. Antisense DNA probes against lncCAMTA1 were designed by Biosearch Probe Designer (1, 5′-CAAACCGCAGATAACGGTCA-3′; 2, 5′-CACGACTGGATAACCGCTTG-3′; 3, 5′-CTAGAGAAGCGACAGTGAGG-3′; 4, 5′-GTGTCTGTATGTTCTTTAGC-3′). Isolated DNA was quantified with SYBR Green PCR Kit (Takara) to measure enrichment of CAMTA1 promoter. The primer sequences for the CAMTA1 promoter were: 5′-CGCTTCTCTGCTTTCGGC-3′ (sense) and 5′-CGTTGTCCCCTTTTCTGGC-3′ (antisense).

### 4.15. Chromatin Immunoprecipitation Assay

The ChIP assay was performed with the EZ-Magna ChIP™ A/G Chromatin Immunoprecipitation Kit (Millipore) using the H3K9ac, H3K27ac, H4ac and H3K4me3 antibodies (Millipore) according to the manufacturer’s protocols. Isolated DNA was quantified with SYBR Green PCR Kit (Takara) to measure enrichment of CAMTA1 promoter. The primer sequences for the CAMTA1 promoter were: 5′-CGCTTCTCTGCTTTCGGC-3′ (sense) and 5′-CGTTGTCCCCTTTTCTGGC-3′ (antisense).

### 4.16. Statistical Analysis

Differences between groups were compared by Student’s *t* test, Wilcoxon signed-rank test, Log-rank test, Mann–Whitney test, or Pearson correlation analysis as indicated. All statistical analyses were performed with SPSS 18.0 (IBM, Chicago, IL, USA). *p* < 0.05 was considered to be significant difference.

## Supplementary Materials

Supplementary materials can be found at www.mdpi.com/1422-0067/17/10/1617/s1.

## References

[1] Jemal A., Bray F., Center M.M., Ferlay J., Ward E., Forman D. (2011). Global cancer statistics. CA Cancer J. Clin..

[2] Laursen L. (2014). A preventable cancer. Nature.

[3] Ahmed F., Perz J.F., Kwong S., Jamison P.M., Friedman C., Bell B.P. (2008). National trends and disparities in the incidence of hepatocellular carcinoma, 1998–2003. Prev. Chronic Dis..

[4] Gores G.J. (2014). Decade in review-hepatocellular carcinoma: HCC-subtypes, stratification and sorafenib. Nat. Rev. Gastroenterol. Hepatol..

[5] Hsu H.T., Wu P.R., Chen C.J., Hsu L.S., Yeh C.M., Hsing M.T., Chiang Y.S., Lai M.T., Yeh K.T. (2014). High cytoplasmic expression of kruppel-like factor 4 is an independent prognostic factor of better survival in hepatocellular carcinoma. Int. J. Mol. Sci..

[6] Ziari K., Zarea M., Gity M., Fayyaz A.F., Yahaghi E., Darian E.K., Hashemian A.M. (2016). Downregulation of miR-148b as biomarker for early detection of hepatocellular carcinoma and may serve as a prognostic marker. Tumour Biol..

[7] Zucchini-Pascal N., Peyre L., Rahmani R. (2013). Crosstalk between beta-catenin and snail in the induction of epithelial to mesenchymal transition in hepatocarcinoma: Role of the ERK1/2 pathway. Int. J. Mol. Sci..

[8] Min L., Ji Y., Bakiri L., Qiu Z., Cen J., Chen X., Chen L., Scheuch H., Zheng H., Qin L. (2012). Liver cancer initiation is controlled by AP-1 through SIRT6-dependent inhibition of survivin. Nat. Cell Biol..

[9] Wang Y., He L., Du Y., Zhu P., Huang G., Luo J., Yan X., Ye B., Li C., Xia P. (2015). The long noncoding RNA lncTCF7 promotes self-renewal of human liver cancer stem cells through activation of Wnt signaling. Cell Stem Cell.

[10] Visvader J.E., Lindeman G.J. (2012). Cancer stem cells: Current status and evolving complexities. Cell Stem Cell.

[11] Wellner U., Schubert J., Burk U.C., Schmalhofer O., Zhu F., Sonntag A., Waldvogel B., Vannier C., Darling D., zur Hausen A. (2009). The EMT-activator ZEB1 promotes tumorigenicity by repressing stemness-inhibiting microRNAs. Nat. Cell Biol..

[12] Liu C., Liu L., Chen X., Cheng J., Zhang H., Shen J., Shan J., Xu Y., Yang Z., Lai M. (2016). Sox9 regulates self-renewal and tumorigenicity by promoting symmetrical cell division of cancer stem cells in hepatocellular carcinoma. Hepatology.

[13] Motawi T.K., El-Boghdady N.A., El-Sayed A.M., Helmy H.S. (2016). Comparative study of the effects of PEGylated interferon-α2a versus 5-fluorouracil on cancer stem cells in a rat model of hepatocellular carcinoma. Tumour Biol..

[14] Haraguchi N., Ishii H., Mimori K., Tanaka F., Ohkuma M., Kim H.M., Akita H., Takiuchi D., Hatano H., Nagano H. (2010). CD13 is a therapeutic target in human liver cancer stem cells. J. Clin. Investig..

[15] Lee T.K., Castilho A., Cheung V.C., Tang K.H., Ma S., Ng I.O. (2011). CD24^+^ Liver Tumor-Initiating Cells Drive Self-Renewal and Tumor Initiation through STAT3-Mediated NANOG Regulation. Cell Stem Cell.

[16] Ma S., Chan K.W., Hu L., Lee T.K., Wo J.Y., Ng I.O., Zheng B.J., Guan X.Y. (2007). Identification and characterization of tumorigenic liver cancer stem/progenitor cells. Gastroenterology.

[17] Easwaran H., Tsai H.C., Baylin S.B. (2014). Cancer epigenetics: Tumor heterogeneity, plasticity of stem-like states, and drug resistance. Mol. Cell.

[18] Chua H.H., Tsuei D.J., Lee P.H., Jeng Y.M., Lu J., Wu J.F., Su D.S., Chen Y.H., Chien C.S., Kao P.C. (2015). RBMY, a novel inhibitor of glycogen synthase kinase 3β, increases tumor stemness and predicts poor prognosis of hepatocellular carcinoma. Hepatology.

[19] Maass P.G., Luft F.C., Bahring S. (2014). Long non-coding RNA in health and disease. J. Mol. Med. (Berl.).

[20] Nie L., Wu H.J., Hsu J.M., Chang S.S., Labaff A.M., Li C.W., Wang Y., Hsu J.L., Hung M.C. (2012). Long non-coding RNAs: Versatile master regulators of gene expression and crucial players in cancer. Am. J. Transl. Res..

[21] Yan X., Hu Z., Feng Y., Hu X., Yuan J., Zhao S.D., Zhang Y., Yang L., Shan W., He Q. (2015). Comprehensive genomic characterization of long non-coding RNAs across human cancers. Cancer Cell.

[22] Yuan J.H., Yang F., Wang F., Ma J.Z., Guo Y.J., Tao Q.F., Liu F., Pan W., Wang T.T., Zhou C.C. (2014). A long noncoding RNA activated by TGF-β promotes the invasion-metastasis cascade in hepatocellular carcinoma. Cancer Cell.

[23] Matouk I., Raveh E., Ohana P., Lail R.A., Gershtain E., Gilon M., de Groot N., Czerniak A., Hochberg A. (2013). The Increasing Complexity of the Oncofetal *H19* Gene Locus: Functional Dissection and Therapeutic Intervention. Int. J. Mol. Sci..

[24] Liu X., Xiao Z.D., Han L., Zhang J., Lee S.W., Wang W., Lee H., Zhuang L., Chen J., Lin H.K. (2016). LncRNA *NBR2* engages a metabolic checkpoint by regulating AMPK under energy stress. Nat. Cell Biol..

[25] Chaudhry M.A. (2013). Expression pattern of small nucleolar RNA host genes and long non-coding RNA in X-rays-treated lymphoblastoid cells. Int. J. Mol. Sci..

[26] Zhang M., Wang W., Li T., Yu X., Zhu Y., Ding F., Li D., Yang T. (2016). Long noncoding RNA SNHG1 predicts a poor prognosis and promotes hepatocellular carcinoma tumorigenesis. Biomed. Pharmacother..

[27] Wang F., Yuan J.H., Wang S.B., Yang F., Yuan S.X., Ye C., Yang N., Zhou W.P., Li W.L., Li W. (2014). Oncofetal long noncoding RNA PVT1 promotes proliferation and stem cell-like property of hepatocellular carcinoma cells by stabilizing NOP2. Hepatology.

[28] Villegas V.E., Zaphiropoulos P.G. (2015). Neighboring gene regulation by antisense long non-coding RNAs. Int. J. Mol. Sci..

[29] Yang F., Xue X., Zheng L., Bi J., Zhou Y., Zhi K., Gu Y., Fang G. (2014). Long non-coding RNA GHET1 promotes gastric carcinoma cell proliferation by increasing c-Myc mRNA stability. FEBS J..

[30] Pandey G.K., Mitra S., Subhash S., Hertwig F., Kanduri M., Mishra K., Fransson S., Ganeshram A., Mondal T., Bandaru S. (2014). The risk-associated long noncoding RNA NBAT-1 controls neuroblastoma progression by regulating cell proliferation and neuronal differentiation. Cancer Cell.

[31] Gupta R.A., Shah N., Wang K.C., Kim J., Horlings H.M., Wong D.J., Tsai M.C., Hung T., Argani P., Rinn J.L. (2010). Long non-coding RNA *HOTAIR* reprograms chromatin state to promote cancer metastasis. Nature.

[32] Prensner J.R., Iyer M.K., Sahu A., Asangani I.A., Cao Q., Patel L., Vergara I.A., Davicioni E., Erho N., Ghadessi M. (2013). The long noncoding RNA SChLAP1 promotes aggressive prostate cancer and antagonizes the SWI/SNF complex. Nat. Genet..

[33] Postepska-Igielska A., Giwojna A., Gasri-Plotnitsky L., Schmitt N., Dold A., Ginsberg D., Grummt I. (2015). LncRNA Khps1 regulates expression of the proto-oncogene SPHK1 via triplex-mediated changes in chromatin structure. Mol. Cell.

[34] Wang X., Sun W., Shen W., Xia M., Chen C., Xiang D., Ning B., Cui X., Li H., Li X. (2016). Long non-coding RNA DILC regulates liver cancer stem cells via IL-6/STAT3 axis. J. Hepatol..

[35] Schraivogel D., Weinmann L., Beier D., Tabatabai G., Eichner A., Zhu J.Y., Anton M., Sixt M., Weller M., Beier C.P. (2011). CAMTA1 is a novel tumour suppressor regulated by miR-9/9* in glioblastoma stem cells. EMBO J..

[36] Henrich K.O., Bauer T., Schulte J., Ehemann V., Deubzer H., Gogolin S., Muth D., Fischer M., Benner A., Konig R. (2011). CAMTA1, a 1p36 Tumor Suppressor Candidate, Inhibits Growth and Activates Differentiation Programs in Neuroblastoma Cells. Cancer Res..

[37] Ha S.Y., Choi I.H., Han J., Choi Y.L., Cho J.H., Lee K.J., Sun J.M. (2014). Pleural epithelioid hemangioendothelioma harboring CAMTA1 rearrangement. Lung Cancer.

[38] McHugh C.A., Chen C.K., Chow A., Surka C.F., Tran C., McDonel P., Pandya-Jones A., Blanco M., Burghard C., Moradian A. (2015). The *Xist* lncRNA interacts directly with SHARP to silence transcription through HDAC3. Nature.

[39] Nio K., Yamashita T., Okada H., Kondo M., Hayashi T., Hara Y., Nomura Y., Zeng S.S., Yoshida M., Sunagozaka H. (2015). Defeating EpCAM^+^ liver cancer stem cells by targeting chromatin remodeling enzyme CHD4 in human hepatocellular carcinoma. J. Hepatol..

[40] Yang X., Ye J., Yan H., Tang Z., Shen J., Zhang J., Yang L. (2016). MiR-491 attenuates cancer stem cells-like properties of hepatocellular carcinoma by inhibition of GIT-1/NF-κB-mediated EMT. Tumour Biol..

[41] Xia P., Wang S., Huang G., Zhu P., Li M., Ye B., Du Y., Fan Z. (2014). WASH is required for the differentiation commitment of hematopoietic stem cells in a c-Myc-dependent manner. J. Exp. Med..

