# Supplementary material for: Long Noncoding RNA lncCAMTA1 Promotes Proliferation and Cancer Stem Cell-Like Properties of Liver Cancer by Inhibiting CAMTA1
Source: Int J Mol Sci. 2016 Sep 23;17(10):1617. doi: 10.3390/ijms17101617 (PMC5085650; doi:10.3390/ijms17101617)
Supplement: Supplementary file 1 [file ijms-17-01617-s001.pdf]

Supplementary Materials: Long Noncoding RNA lncCAMTA1 Promotes Proliferation and Cancer Stem Cell-Like Properties of Liver Cancer by Inhibiting CAMTA1

Li-Juan Ding, Yan Li, Shu-Dong Wang, Xin-Sen Wang, Fang Fang, Wei-Yao Wang, Peng Lv, Dong-Hai Zhao, Feng Wei and Ling Qi

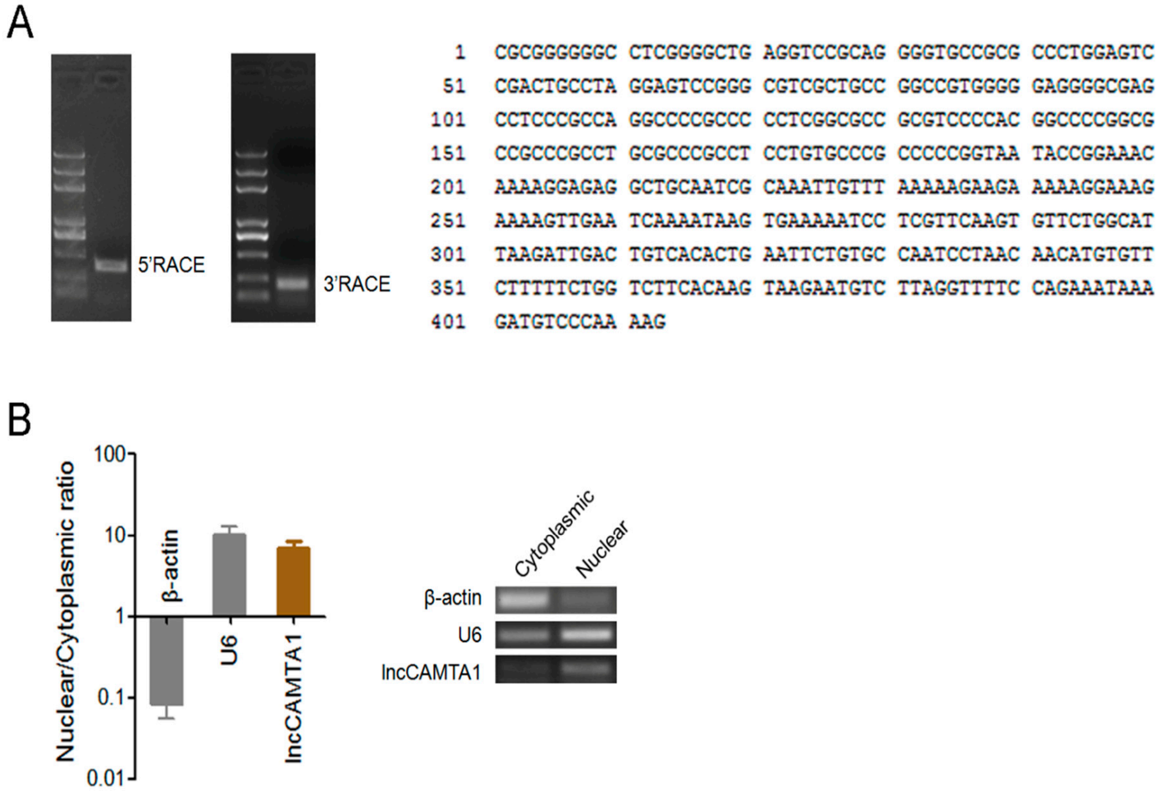

Figure S1. Full-length sequences and subcellular localization of lncCAMTA1 in hepatocellular carcinoma cells. (**A**) Full-length nucleotide sequences of lncCAMTA1; (**B**) Quantitative real-time PCR (qRT-PCR) analysis of lncCAMTA1 subcellular location in HCCLM3 cells. β-actin and U6 were used as cytoplasmic and nuclear markers respectively. $n = 3$.
